# Editorial: Computational drug discovery of medicinal compounds for cancer management, volume II

**DOI:** 10.3389/fchem.2024.1446510

**Published:** 2024-06-24

**Authors:** Khurshid Ahmad, Sibhghatulla Shaikh, Faez Iqbal Khan, Mohammad Ehtisham Khan

**Affiliations:** ^1^ Department of Medical Biotechnology, Yeungnam University, Gyeongsan, Republic of Korea; ^2^ Department of Biological Sciences, School of Science, Xi’an Jiaotong-Liverpool University, Suzhou, Jiangsu, China; ^3^ Department of Chemical Engineering Technology, College of Applied Industrial Technology, Jazan University, Jazan, Saudi Arabia

**Keywords:** computational methods, drug discovery, cancer, medicinal compounds, druglikeness, artificial intelligence

Cancer, one of the leading causes of death worldwide, remains a serious public health concern. Even with significant advances in drug discovery, developing novel and useful small-molecule drugs remains a difficult, costly, and time-consuming process that necessitates the collaboration of numerous experts in interdisciplinary domains such as drug metabolism, computational biology, and clinical research. Computational approaches in drug discovery are crucial and in high demand, significantly impacting time and cost savings while increasing efficiency.

Computational approaches, such as molecular docking, molecular dynamics simulations, pharmacophore modeling, and QSAR, are efficient tools for gaining insights into the structure-function relationships of small molecules and medicinal compounds with target proteins. These methods are widely used in the identification and optimization of lead compounds. The key objectives of the drug discovery process are to identify potential toxicity, predict the metabolic fate of a drug candidate, and establish a connection between pharmacodynamics and pharmacokinetics. Recent advancements in *in silico* techniques have enabled researchers to collect more reliable data. Additionally, the rise of AI and deep learning in drug discovery is revolutionizing the field, significantly impacting the discovery of new molecules for various diseases, including cancer.

Researchers were interested in this Research Topic, and many manuscripts were submitted. Six Original Research articles that explain advanced *in silico* techniques relevant to the drug discovery domain and span a broad spectrum of CADD topics have been published out of these submissions. A brief overview of the articles that have been published is as follows:

Rahaman et al. investigated the mechanism of plumbagin’s (PLM) binding to calf thymus DNA. UV-Vis spectroscopy indicated that PLM binds to ctDNA, and dye displacement assays confirmed the formation of PLM-ctDNA complex. PLM has little effect on the structural integrity of ctDNA, as evidenced by minimal changes in circular dichroism spectra, implying peripheral rather than intercalative binding. Measurements of relative viscosity and minimal changes in DNA melting temperature further supported PLM’s groove binding nature. In silico analysis, identified PLM as a minor groove binder and MD simulation demonstrated the formation of a stable complex. This study elucidates into the cytotoxic and genotoxic mechanisms of PLM, emphasizing its potential as a cancer treatment.

Zafar et al. identified tannins, saponins, steroids, and cardiac glycosides in *Nigella sativa* leaves, with alkaloid and saponin levels of 9.4% ± 0.04% and 1.9% ± 0.05%, respectively. The methanol extract demonstrated the highest yield and antioxidant profile, with Total Flavonoid Content at 127.51 ± 0.76 mg/100 g and Total Phenolic Content at 134.39 ± 0.589 mg GAE/100 g. The Total Antioxidant Capacity (TAC) assay revealed a concentration-dependent effect. Hemolysis testing revealed varying degrees of hemolysis (3.7%–16.5%), emphasizing the importance of careful extraction and dosage. In silico studies demonstrated Neuropilins’ potential as anticancer agents, with a particular focus on the PI3K protein. These findings indicate that *N. sativa* leaves have significant health benefits and have the potential to be used in the development of targeted cancer therapies.

Hosen et al. found potential inhibitors of the staphylococcal accessory regulator protein in Santalum album-derived phytochemicals. Vitexin, isovitexin, and orientin had the highest binding energies: −9.4 kcal/mol, −9.0 kcal/mol, and −8.6 kcal/mol, respectively. Molecular dynamics simulations demonstrated that the docked complexes were stable. ADMET profiles and AMES tests showed that these compounds were non-toxic. *In vitro* tests revealed significant inhibitory activity against *Staphylococcus aureus*. These findings imply that these compounds could act as natural leads for inhibiting *S. aureus* pathogenesis and could be used in antibiotic therapy for *S. aureus*-associated skin cancer.

Alzain et al. developed a structure-based pharmacophore model and screened 449,008 natural products (NPs) from the SN3 database for compounds with pharmacophoric properties similar to the native ligand. This screening identified 650 compounds, which were then subjected to molecular docking and binding free energy calculations. SN0021307, SN0449787, and SN0079231 had lower binding free energies (−57.12, −49.81, and −46.05 kcal/mol, respectively) than the reference ligand (−37.75 kcal/mol), indicating a stronger binding affinity. Molecular dynamics simulations revealed the stability of the ligand-receptor complexes. These findings indicate that SN0021307, SN0449787, and SN0079231 have significant potential as Pin1 inhibitors and warrant further investigation for their efficacy and safety in cancer treatment.

Rizvi et al. investigated the anti-cancer properties of garcinol in Glioblastoma multiforme (GBM). At a concentration of 30 μM, garcinol decreased the viability of rat glioblastoma C6 cells and caused nuclear fragmentation and condensation. It caused a dose-dependent loss of mitochondrial membrane potential, resulting in caspase activation. Garcinol inhibited NF-κB and reduced the expression of survival genes (Bcl-XL, Bcl-2, and survivin) and proliferation genes (cyclin D1). Molecular docking analysis revealed interactions between garcinol and NF-κB involving key amino acid residues (Pro275, Trp258, Glu225, and Gly259). Garcinol inhibits NF-κB activity, leading to apoptotic cell death in C6 cells, indicating potential as a therapeutic agent for GBM.

Zarougui et al. used molecular modeling techniques to predict new potential breast cancer drugs, with a focus on identifying inhibitors of the MCF-7 cell line. They employed a variety of computational techniques, including the 3D-QSAR method, which resulted in two robust models that were externally validated and aided in identifying functional groups with improved inhibitory abilities. ADMET research and pharmacokinetics guided the development of six inhibitors, two of which showed strong interactions with the target protein in molecular docking simulations. MD simulations and binding free energy calculations confirmed these findings. The study provides important information for the development of novel breast cancer inhibitors with promising therapeutic applications.

Finally, we hope that the articles published in this Research Topic will highlight advancements in the use of computational methodologies to facilitate the design of novel compounds directed at various protein targets involved in cancer management. Furthermore, it is expected that these articles will contribute to a more in-depth and comprehensive understanding of the complex biological processes that underpin cancer, potentially leading to new therapeutic interventions. We hope that these articles will inspire, inform, and guide researchers and scholars working in this field.

